# Interstitial fluid pressure, vascularity and metastasis in ectopic, orthotopic and spontaneous tumours

**DOI:** 10.1186/1471-2407-8-2

**Published:** 2008-01-07

**Authors:** Sarah Jane Lunt, Tuula MK Kalliomaki, Allison Brown, Victor X Yang, Michael Milosevic, Richard P Hill

**Affiliations:** 1 Ontario Cancer Institute, Princess Margaret Hospital, University of Toronto, 610 University Ave, Toronto, Ontario, M5G 2M9, Canada; 2 Radiation Medicine Program, Princess Margaret Hospital, University of Toronto, 610 University Ave, Toronto, Ontario, M5G 2M9, Canada; 3 Department of Medical Biophysics, Princess Margaret Hospital, University of Toronto, 610 University Ave, Toronto, Ontario, M5G 2M9, Canada; 4 Department of Radiation Oncology, Princess Margaret Hospital, University of Toronto, 610 University Ave, Toronto, Ontario, M5G 2M9, Canada; 5 Division of Neurosurgery, Princess Margaret Hospital, University of Toronto, 610 University Ave, Toronto, Ontario, M5G 2M9, Canada

## Abstract

**Background:**

High tumour interstitial fluid pressure (IFP) has been adversely linked to poor drug uptake in patients, and to treatment response following radiotherapy in cervix cancer patients. In this study we measured IFP values in a selection of murine and xenograft models, spontaneously arising or transplanted either intramuscularly (i/m) or orthotopically and analysed their relationship to tumour vascularity and metastatic spread.

**Methods:**

KHT-C murine fibrosarcoma, ME180 and SiHa human cervix carcinoma were grown either intramuscularly (i/m), sub-cutaneously (s/c) or orthotopically. Polyoma middle-T (MMTV-PyMT) transgenic spontaneous mammary tumours were studied either as spontaneous tumours or following orthotopic or i/m transplantation. IFP was measured in all tumours using the wick-in-needle method. Spontaneous metastasis formation in the lungs or lymph nodes was assessed in all models. An immunohistochemical analysis of tumour hypoxia, vascular density, lymphatic vascular density and proliferation was carried out in ME180 tumours grown both i/m and orthotopically. Blood flow was also assessed in the ME180 model using high-frequency micro-ultrasound functional imaging.

**Results:**

Tumour IFP was heterogeneous in all the models irrespective of growth site: **KHT-C **i/m: 2–42 mmHg, s/c: 1–14 mmHg, **ME180**: i/m 5–68 mmHg, cervix 4–21 mmHg, **SiHa**: i/m 20–56 mmHg, cervix 2–26 mmHg, **MMTV-PyMT**: i/m: 13–45 mmHg, spontaneous 2–20 mmHg and transplanted 2–22 mmHg. Additionally, there was significant variation between individual tumours growing in the same mouse, and there was no correlation between donor and recipient tumour IFP values. Metastatic dissemination to the lungs or lymph nodes demonstrated no correlation with tumour IFP. Tumour hypoxia, proliferation, and lymphatic or blood vessel density also showed no relationship with tumour IFP. Speckle variance analysis of ultrasound images showed no differences in vascular perfusion between ME180 tumours grown i/m versus orthotopically despite differences in IFP.

**Conclusion:**

Our studies across a range of different tumour models showed substantial heterogeneity in tumour IFP, suggesting differences in the vascular development and interstitial fluid dynamics in the individual tumours. The results demonstrate a strong stochastic aspect to tumour IFP development, notably the variation apparent between different tumours within the same animal and the lack of correlation between donor and recipient tumours.

## Background

Solid tumours show interstitial fluid pressures (IFP) that are elevated above that of normal tissues. Tumour growth and development is supported by both the pre-existing host vasculature and by neovasculature generated through the process of angiogenesis. Tumour angiogenesis generates abnormal vessels [1-3] that demonstrate several anomalies including an incomplete or absent endothelial cell layer and basement membrane which makes them hyper-permeable [4]. These vessels exhibit a high resistance to capillary blood flow and a low resistance to transcapillary flow, resulting in a net efflux of fluid into the surrounding interstitial space where a lack of functional lymphatics allows it to accumulate, distending the elastic extracellular matrix and increasing the interstitial pressure [5,6]. An equilibrium is established where the capillary and interstitial pressures are equivalent resulting in reduced fluid movement through the interstitium [6]. In addition, the tumour interstitium itself is thought to be abnormal, comprising a dense network of collagen fibres, as well as increased fibroblasts, macrophages and other cells involved in inflammation, which further contribute to elevated IFP values [3]. It is clear from these previous studies that high IFP in tumours arises because of the complex interplay between the abnormal vasculature and the abnormal interstitium. However, the pathophysiologic mechanisms underlying widely varying IFP values in human and experimental tumours of the same and differing types, and the influence of growth site and the host, is less well understood.

Elevated tumour IFP plays a role in the pathophysiological microenvironment that characterises solid tumours contributing to disease progression and therapeutic resistance [3,6-9]. The mechanisms involved remain to be fully elucidated but several experimental animal studies have shown an improved uptake of therapeutic agents in response to a reduction in tumour IFP suggesting that high tumour IFP acts as a barrier to drug delivery [10-17]. Furthermore, there are clinical data showing that tumour IFP correlates with response to treatment [18,19], with strong evidence for high IFP as an adverse prognostic indicator in cervix cancer patients treated with radiotherapy [7,9]. Patients in the latter study were significantly more likely to develop distant recurrence if they presented with a tumour IFP value above the group median (19 mmHg), which suggests a role for IFP in metastatic spread. A relationship has also been observed between tumour IFP values and metastasis in experimental melanoma xenografts [20].

These data, coupled with the breadth of data demonstrating elevated IFP in a wide range of human tumours [7,18,19,21-25], designate high tumour IFP as an important therapeutic problem. Further preclinical investigation is needed to understand the mechanisms underlying the adverse prognostic effect of high IFP and the implications for treatment. However, little is known about the most appropriate experimental model for these studies in relation to clinical tumour behaviour. To date most studies have focused on tumour models grown sub-cutaneously [13,26-28] although evidence suggests that orthotopic models may be more clinically relevant [29,30]. Although IFP has been measured in a variety of different tumour models, to our knowledge no previous study has focussed on the influence of tumour growth site on the development of the pathophysiological tumour microenvironment, or more specifically, tumour IFP. As such, the purpose of this study was to assess IFP in a number of different murine (KHT-C) and xenograft (ME180, SiHa) tumour models growing both ectopically and orthotopically and to examine features of the tumours (lymphatic and blood vascular density, hypoxia, perfusion) that might relate to the IFP levels and to disease progression. The murine and xenograft models were selected on the basis of previous studies within our lab [31,32] and the polyoma middle-T (MMTV-PyMT) transgenic spontaneous mammary tumour model was included to allow comparison with the transplanted models [33]; IFP has only previously been assessed in one other spontaneously arising tumour model [34]. The orthotopic human cervix cancer xenograft models were included because of the direct relevance of this model to our clinical program [7,9]. IFP was examined for each of the different tumour models and growth sites and related to tumour size, metastatic dissemination, tumour hypoxia, proliferation and vascular and lymphatic density.

## Methods

### Mice and tumour cell lines

Experiments were performed using MMTV-polyoma middle-T transgenic mice (MMTV-PyMT;[33]) bred in-house, the previously described KHT-C murine fibrosarcoma cell line [35] and the ME180 and SiHa human cervical carcinoma cell lines stably transfected to constitutively express the fluorescent marker DsRed [36]. All cell lines were maintained on an alternative *in vitro*/*in vivo *growth cycle. *In vitro *cells were maintained as monolayers in plastic tissue culture flasks using α-MEM medium (Life Technologies, Inc., Burlington, Canada) supplemented with 10% fetal bovine serum (Wisent, Quebec, Canada). The cervical carcinoma cell lines were maintained under G-418 selection (400 μg/ml). Cells between their 2^nd ^and 5^th ^*in vitro *passage were removed from the flasks during exponential growth using 0.05% trypsin for transplantation into mice. KHT-C cells were transplanted into syngeneic 8–12 week old C3H/HeJ male mice (Jackson Laboratory, Bar Harbour, ME). ME180 and SiHa cells were transplanted into 8–12 week old female CB-17/SCID mice obtained from an in-house breeding program. PyMT cells were transplanted into female FVB (wild-type, w.t.) or SCID mice. Tumours were initiated either intramuscularly (i/m) in the left gastrocnemius muscle, or sub-cutaneously (s/c) on the flank by injection of 2.5 × 10^5 ^or 5 × 10^5 ^cells respectively in a 50 μl volume of α-MEM media. Tumours growing i/m were monitored by measuring the external leg diameter of the mouse. Tumours growing s/c were measured directly. Orthotopic cervical and mammary gland tumours were initiated from donor tumours using protocols described below. Animals were housed at the Ontario Cancer Institute animal facility and had access to food and water *ad libitum*. All experiments were performed under protocols approved according to the regulations of the Canadian Council on Animal Care.

### Orthotopic implantation in the cervix or mammary gland

The method for orthotopic implantation of tumour fragments into the cervix has been described in detail previously [36]. In brief, donor tumours grown i/m were excised under sterile conditions and small fragments (1.5–2 mm in diameter) were sutured into the site of a small incision in the uterus at the level of the cervix. Once tumours were palpable, IFP measurements were taken and tumours, lumbar lymph nodes and lungs were imaged/removed for further analysis. Spontaneously arising donor tumours in MMTV-PyMT transgenic mice were excised under sterile conditions and divided into small fragments of approximately 2 mm in diameter. A fine incision was made in the skin to expose the 4^th ^mammary gland. A small incision was made in the fat pad of the 4^th ^mammary glands and a tumour fragment was sutured in place using a single 8-0 silk suture. The skin was closed using stainless steel wound clips. The left and right 4^th ^mammary glands were implanted with a donor tumour fragment from either the same or a different donor tumour (Additional File 2). This allowed the effect of donor tumour variability on the subsequent development of the recipient tumour microenvironment to be assessed without the confounding factor of inter-animal variability. All surgical procedures were carried out under anaesthesia (2% isofluorane). Buprenorphine (0.1 mg/kg) was administered s/c following surgery to alleviate pain.

Tumour growth was monitored using callipers to measure the width and length of the tumour; once a size of 50–80 mm^2 ^was attained IFP measurements were initiated. Mice were sacrificed once a tumour reached a size of 200–250 mm^2^. Half of each tumour was fixed in 10% neutral buffered formalin and half was snap frozen in OCT for histological analyses. The lungs were excised for examination of metastases.

### Pressure treatment *in vitro*

KHT-C tumour cells were exposed to elevated pressure (20 mmHg) for various times *in vitro *in 10 cm tissue culture dishes seeded with a sub-confluent cell monolayer in a modular incubator chamber (Billups-Rothenberg, Del Mar, Canada). Pressure levels used were based on the average IFP values apparent when grown i/m. Pressure was controlled through adjustments of a dual scale low pressure gauge (0–15 KPa; Cole Parmer, Quebec, Canada) on the outlet port. Control cells were gassed using the same system, but without the addition of a pressure gauge on the outlet port, allowing the gas to flush through freely at atmospheric pressure. The metastatic potential of pressure treated tumour cells was assessed using an experimental lung metastases assay [37].

### IFP measurements

Interstitial fluid pressure was measured using a wick-in-needle technique [38]. Measurements were made using a 23-gauge needle with side port connected to a pressure transducer (Model P23XL, Viggo-Spectramed, Oxnard, CA) and an electronic data acquisition and recording system (Model MP100, World Precision Instruments, Sarasota, FL) through 470 mm of PE20 polyethylene tubing (Becton Dickinson, Franklin Lakes, NJ, USA). A "wick" was placed in the distal portion of the needle, and the entire system was flushed with a heparin sulphate/saline solution (1:10) [39]. IFP measurements were taken at three to four different locations in the tumour, and the mean value of these readings was taken to represent the tumour IFP.

### Assessment of macroscopic and microscopic metastases

Following sacrifice of tumour bearing animals, the lungs were removed and fixed overnight in Bouin's solution (BDH Inc., Toronto, Canada). A dissecting microscope was used to count the number of visible metastases in each of the five lobes and the total number of lesions counted per lung reported (KHT-C and MMTV-PyMT). In the case of too many metastatic lesions to count, the wet weight of the lungs was taken as representative of metastatic burden. For the orthotopic ME180 tumours that had been transfected to express DsRed, lymph node metastases were visualised by fluorescence and counted as previously described [36].

Microscopic lung metastases were detected immunohistochemically (ME180 and SiHa) following fixation of lungs in Bouin's solution (BDH Inc., Toronto, Canada). All five lobes were paraffin embedded and four 4 μm sections 150 μm apart were cut from each lobe. The number of visible micro-metastases in each of the five lobes was then counted using a light microscope. Two or more clumped tumour cells were scored as a lesion. Micro-metastases are reported as the total number of lesions counted per lung.

### Histological Analyses

Analyses were carried out using immunohistochemistry for tumour hypoxia (EF5), vascular density (CD31), lymphatic vessel density (LYVE-1), and proliferation (Ki67). Tumour bearing animals were injected with the hypoxia marker EF5 [2-(2-nitro-1*H*-imidazole-1-yl)-*N*-(2,2,3,3,3-pentafluoropropyl) acetamide]; obtained from Dr. Cameron Koch, University of Pennsylvania, at 10 mg/kg 2.5 h prior to tumour excision. Once excised, half of the tumour was fixed in 10% neutral buffered formalin, and the remaining half placed in optimal cutting temperature (OCT) embedding medium (Tissue-Tek, Sakura, USA), and snap frozen in liquid nitrogen. Paraffin-embedded tissue was used for all markers with the exception of CD31.

For each marker, two sections were cut 100 μm apart due to intra-tumoural heterogeneity (4 μm sections for paraffin-embedded tissue, 5 μm sections for frozen tissue). The slides were then processed according to standard immunohistochemical protocols. The primary antibodies used were: for EF5, the biotinylated antibody ELK3-51 (1/500; a gift from Dr. Cameron Koch, University of Pennsylvania); for CD31, the rat anti-mouse CD31, clone MEC 13.3 (1/500; Pharmingen, Canada); for Ki67, mouse anti-human Ki67 clone MIB-1 (1/100; DAKO, Canada); for LYVE-1, rabbit anti-mouse LYVE-1 (1/200 Abcam, Canada). For all markers, apart from EF5, primary incubation was followed by a 30 minute incubation with a biotinylated secondary (Vector Labs, Canada) and horseradish peroxidase conjugated ultrastreptavidin labelling reagent (ID labs, Canada). Nova Red (Vector) with Mayer's hematoxylin counterstain was used for chromogenic detection.

The stained sections were analysed using the Aperio imaging system (Aperio Technologies, California). Entire sections were scanned using the ScanScope CS and the total area of positive staining quantified using a positive pixel algorithm designed for brown/blue immunohistochemical stains. The same settings were used for each stain across all images, and the area of positive staining was calculated by dividing the total number of positive pixels (weak, medium and strong staining) by the total number of pixels in the image (positive + negative pixels) to yield the overall percentage of positive pixels. To assess intra-tumour heterogeneity for each stain, 10 (CD31) or 20 (EF5, Ki67 and LYVE1) high-magnification (20×) fields were randomly selected from each tumour section and analysed independently using the same methodology.

### Blood flow detection using high-frequency micro-ultrasound functional imaging

Real-time ultrasound biomicroscopic imaging of anaesthetised (2% isofluorane) mice was carried out using speckle variance analysis of high frequency ultrasound (Vevo660, VisualSonics, Inc., Toronto, ON, Canada) images as previously described [40]. The ultrasound transducer transmits at a central frequency of 40 MHz with a focal length of 6 mm. The lateral and axial resolutions were 68 and 38 μm, respectively. Ultrasound gel (Aquasonic 100, Parker Laboratories, Fairfield, NJ) was used as a coupling agent on the skin. A minimum of five brightness mode (B mode) two-dimensional image planes were acquired per tumour, each with cineloops of 300 frames at a frame rate of 17/s. Speckle pattern and intensity during real-time B mode imaging of stationary tissue remain constant, and the temporal variance of speckle intensity increases with tissue motion. A speckle-variance flow-processing algorithm devised by Yang [41] was used to calculate changes in speckle intensity between sequential frames as an indication of functional blood flow [40]. This technique gives a relative indication of the number of perfused vessels in each tissue plane, and has been validated previously against perfusion assessed using injection of Hoechst 33342 [40]. Each of the 5 B mode image planes were analysed for 3 i/m and 6 cervix ME180 tumours.

### Statistical Analysis

Experiments with three or more groups were analysed for statistical significance using the Kruskal-Wallis statistic, and individual comparisons within these groups were carried out using Dunn's test. Experiments with two groups were analysed for statistical significance using the Mann-Whitney statistical test. Correlation was assessed using the correlation coefficient derived from linear-regression analysis.

## Results

### IFP varies between tumours of both the same and different origins

**Figure 1:**
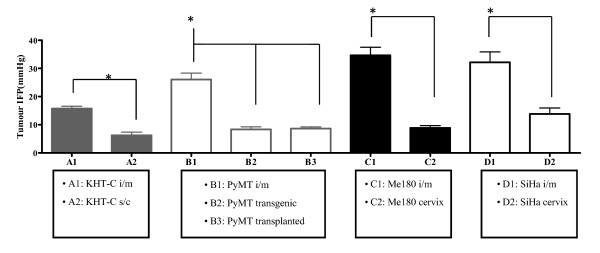
**Comparison of IFP values between different growth sites and different models**. Mean IFP is shown for each tumour line growing orthotopically and ectopically; KHT-C i/m (n = 104) and s/c (n = 12) tumours, MMTV-PyMT i/m (n = 15), transgenic spontaneous mammary gland (n = 27), and transplanted orthotopic mammary gland (n = 62) tumours, ME180 i/m (n = 26) and orthotopic cervix (n = 28) tumours, SiHa i/m (n = 9) and orthotopic cervix (n = 10) tumours. The error bars represent the standard error of the mean. An asterix indicates that a group is significantly (p ≤ 0.05) different to the other groups.

Tumour IFP was measured in a number of different murine and xenograft tumour models grown both ectopically and orthotopically. Substantial inter-tumour (between individual tumours) heterogeneity was apparent for all tumour models irrespective of site (Figure 1 and Additional File 1). Interestingly, ME180 and SiHa tumours grown orthotopically in the cervix or MMTV-PyMT tumours in the mammary fat pad consistently demonstrated IFP values lower than those grown i/m (p < 0.05). Similarly, KHT-C growing s/c had lower IFP values as compared to tumours growing in the i/m site, demonstrating that in these models tumour IFP is higher when growing intra-muscularly. The least inter-tumoural variability in the i/m site was seen in the KHT-C tumour model. In the cervical carcinoma xenograft models growing orthotopically, IFP was significantly higher in the SiHa model as compared to the ME180 model.

**Figure 2:**
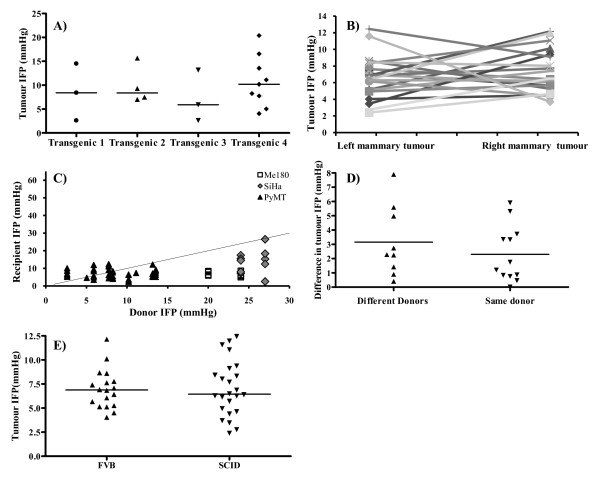
**Inter-tumour variability within the same animal**. **A) **The mean IFP value (mmHg) for each individual mammary gland tumour arising in four different MMTV-PyMT transgenic mice is shown. The bar represents the median IFP value (mmHg) for each mouse. There was substantial variation between different tumours within the same mouse. **B) **The mean IFP value (mmHg) for each of two orthotopic MMTV-PyMT tumours, one transplanted into the left and one into the right 4^th ^mammary gland, is shown. The tumours from the same donor animal are connected by a solid line. Again, there was obvious variation in the IFP values of different tumours growing within the same animal. **C) **Recipient tumour IFP (mmHg; y-axis) plotted as a function of the mean donor IFP (mmHg; x-axis). The line of identity is shown. There was no correlation between donor and recipient IFP. **D) **The difference in the mean IFP values of two different tumours growing in the left and the right mammary glands is shown, grouped according to whether the two tumours were initiated from the same or different donor tumours. The median IFP for each group is indicated; there was no significant difference (Mann Whitney test p ≥ 0.05). **E) **The mean tumour IFP is shown for each individual MMTV-PyMT mammary gland tumour transplanted into either SCID or FVB mouse backgrounds. The bar indicates the median IFP values. The IFP values were comparable for both mouse backgrounds.

The inter-tumour heterogeneity apparent in tumours growing at the same site in different animals was also observed in individual tumours growing within the same animal (Figure 2). Multiple spontaneously arising tumours in the mammary glands of an individual transgenic MMTV-PyMT mouse demonstrated a wide range of IFP values (5–20 mmHg, Figure 2A). Similarly, when mammary tumours derived from the same donors were directly transplanted orthotopically into both the left and right 4^th ^mammary gland of recipient mice, the subsequent tumours showed divergent IFP values (Figure 2B). However, the range of IFP values seen in spontaneously arising mammary gland tumours was comparable to the range of values seen in tumours transplanted orthotopically (Additional File 1).

To investigate whether transplant into different host mice impacted IFP levels, mammary gland tumours were transplanted into the mammary glands of either SCID (immune deficient) or FVB (syngeneic) recipient mice. Tumour IFP values were found to be similar irrespective of the mouse background (Figure 2E), suggesting that the tumour cell genetics may have more impact on the range of IFP values for a specific tumour type than the host genetics in experimental models.

### Donor tumour IFP is not predictive of recipient tumour IFP

To ascertain whether the pre-existing tumour microenvironment and molecular interactions can impact on tumour development, donor tumour IFP values were measured prior to transplantation into the relevant orthotopic site in the ME180, SiHa and MMTV-PyMT tumours. IFP values in the transplanted tumours showed no correlation with those in the donor tumours (Figure 2C). Although the donor ME180 and SiHa tumours were grown i/m prior to orthotropic transplantation, which might have confounded this comparison, the donor PyMT tumours were grown orthotopically in the mammary fat pad. Furthermore, the mice bearing two MMTV-PyMT orthotopically transplanted mammary tumours showed disparities in tumour IFP measurements irrespective of whether they were initiated from different or the same donors (Additional File 2 & Figure 2D). All these results are consistent with no relationship between IFP values in the donor and transplanted tumour irrespective of transplantation site.

### Correlation between tumour size and IFP is tumour specific

**Figure 3:**
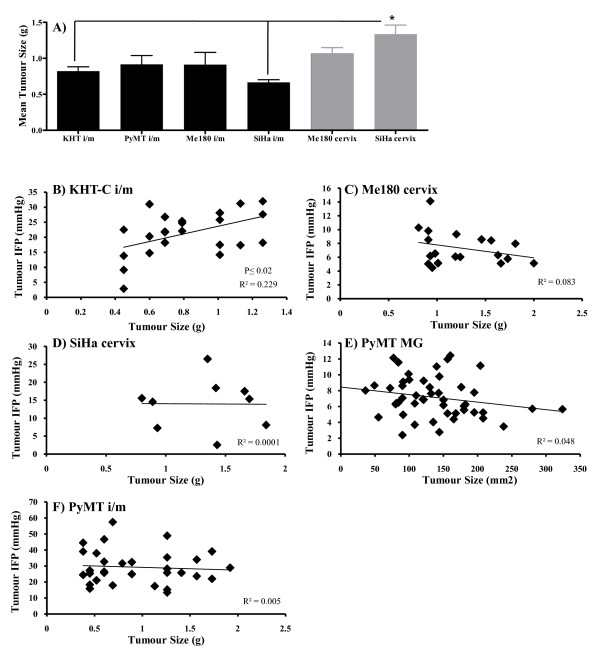
**Tumour size and IFP values**. Graph **A **shows the mean tumour size at the time of IFP measurement for each model. Error bars represent standard error of the mean, and a significant difference is indicated by an asterix. There were no significant differences in mean tumour size between models, with the exception of SiHa tumours growing in the cervix, which were significantly larger than SiHa or KHT-C tumours growing i/m. Graphs **B-F **show the mean tumour IFP (mmHg, y-axis) of individual tumours plotted against the tumour size (g or mm^2^; x-axis). The R^2 ^value is shown on each graph. **B) **KHT-i/m tumours, **C) **ME180 cervix tumours, **D) **SiHa cervix tumours, **E) **MMTV-PyMT transplanted orthotopic mammary gland tumours, **F) **MMTV-PyMT i/m tumours. There was a correlation between tumour size and IFP only in the KHT-C tumour model growing i/m.

Previous studies have noted a tumour specific correlation between tumour size and IFP [7,26,28,42-45]. Although, the mean tumour size at the time of the IFP measurements was similar for all of the tumour models at all sites (Figure 3A), the use of tumours with different sizes permitted an analysis of the relationship between tumour size and IFP in a subset of tumours within most of the models (Figure 3B–F). A significant correlation between tumour size and IFP was seen only for KHT-C tumours growing i/m (p ≤ 0.02; Figure 3B).

### High tumour IFP does not correlate with increased metastatic efficiency

**Figure 4:**
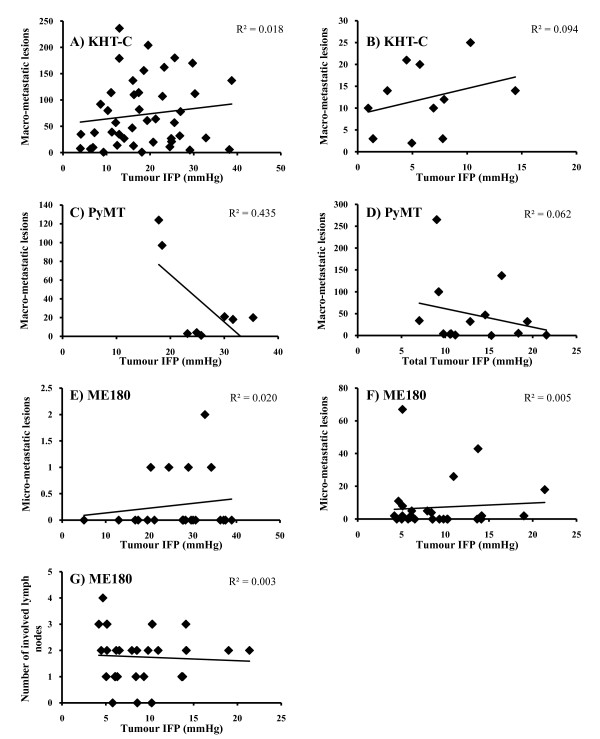
**Metastases presentation as a function of tumour IFP**. The number of macro- or microscopic lung lesions or involved lymph nodes counted is shown for different tumour models. The data is presented as number of metastastic lesions/involved lymph nodes (y-axis) of individual tumours plotted against mean tumour IFP (mmHg; x-axis). The R^2 ^value is shown on each graph. There was no significant correlation for any of the models. **A) **KHT-C i/m, **B) **KHT-C s/c tumours, **C) **MMTV-PyMT i/m tumours,**D) **MMTV-PyMT MG tumours (total mean IFP shown), **E) **ME180 i/m tumours, **F) **ME180 cervix tumours (lung metastases), **G) **ME180 cervix tumours (lymph node metastases).

Cervical carcinoma patients with a high tumour IFP are at increased risk of distant recurrence after treatment with radiotherapy [7,9]. Furthermore, Rofstad et al (2002) [20] showed experimentally that oxic melanoma tumours with a high IFP showed a significantly increased metastatic ability. However, the relationship between tumour IFP and metastatic disease has not been widely studied. Consequently, we examined metastases formation in each of the tumour models (apart from the SiHa model which did not metastasise from either the orthotopic or i/m site) but found no correlation between a high tumour IFP and enhanced metastatic potential (Figure 4A–G). For the MMTV-PyMT model growing orthotopically or in the transgenic animals, due to the presence of multiple tumours with divergent IFP values in each animal, metastases data are plotted according to the mean IFP value of all the tumours within that animal.

Elevated tumour IFP is a consequence of complex pathophysiologic interactions between the tumour vasculature and the interstitium. Factors that influence IFP may also influence other aspects of the tumour microenvironment, including the development of hypoxia, which has previously been shown to enhance metastatic ability [31,32,37,46-50] and could potentially confound the *in vivo *analysis. Thus, in an attempt to elucidate the role of elevated pressure *per se*, KHT-C tumour cells were also grown under conditions of high pressure *in vitro *prior to analysis of metastatic ability with an experimental metastases assay *in vivo*. No evidence for a correlation between exposure to elevated pressure and metastatic ability was observed for the KHT-C tumour line (Additional File 3), consistent with the spontaneous metastases data. The other models were not investigated for this study, as they do not form a sufficient number of lung metastases following i/v injection.

### Model-specific differences in vascular density, tumour hypoxia and tumour proliferation in relation to tumour IFP

Differences in IFP from one tumour to the next may be due to differences in the underlying tumour microvascular and lymphatic architecture. Consequently, an immunohistochemical analysis was carried out using CD31 (vascular density) and LYVE-1 (lymphatic vessel density) staining. In addition, proliferation was assessed using the proliferation marker Ki67, as it has been suggested that genes involved in cellular proliferation may be upregulated in tumours with higher IFP values [51,52]. Tumour hypoxia, a common feature of the pathophysiological tumour microenvironment, was also examined using the hypoxic marker EF5.

**Figure 5:**
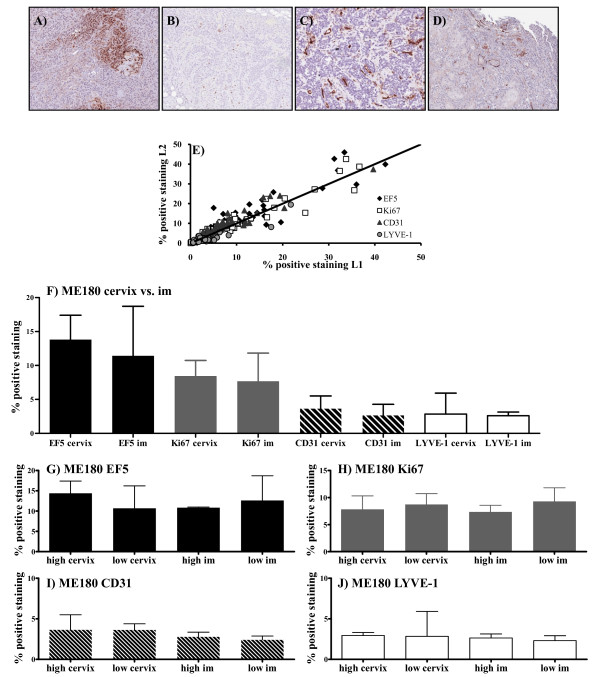
**Immunohistochemical analyses of tumour hypoxia, proliferation and vascular area**. A representative image for each marker is shown (10× magnification; ME180 tumour growing i/m) **A) **EF-5,**B) **Ki67,**C) **CD31,**D) **LYVE-1.**E) **Sections were taken on two levels, 100 μm apart to assess intra-tumoural heterogeneity. The correlations between levels 1 (L1) and 2 (L2) are shown. Each point represents an individual tumour (includes data from PyMT tumours; see Additional File 5), and the legend indicates the tumour model and site of growth. The line of identity is shown. Quantitative analysis of two sections from each tumour; bars represent the median percentage of positive staining [(positive pixels/total number of pixels (positive + negative)) × 100] and error bars show the range. **F) **Each marker according to whether the tumour was grown in the cervix (n = 9) or i/m (n = 9). **G-J) **Each marker in both growth sites split according to whether the tumour IFP was above (high; n = 5) or below (low; n = 5) the median IFP; **F) **EF-5, **G) **Ki67, **H) **CD31 and **I) **LYVE-1.

The ME180 model growing either i/m or orthotopically was chosen for detailed characterisation, because this model is the most appropriate for an analysis of metastatic progression to the regional lymph nodes, the most clinically relevant metastatic site, as well as the lungs. In addition, previous studies have demonstrated that exposure of ME180 orthotopic tumour bearing mice to cyclic hypoxia enhances metastatic spread to the lymph nodes [31]. Two tumour sections taken 100 μm apart showed a good agreement for all of the markers (Figure 5E). The analysis of these sections indicated no significant differences in tumour hypoxia, proliferation, vascular area or lymphatic vascular area between ME180 tumours growing in the i/m site versus orthotopically. Furthermore, there were no significant differences between tumours with a high (above median) or low (below median) IFP for either site (Figure 5F–J). To assess intra-tumour variability, a random high-power field (20×) analysis was carried out for each parameter. The average values for all fields were analogous to the values obtained through whole image analysis. There were no significant differences between the tumours growing i/m versus orthotopically (Additional File 4A). Furthermore, although there was substantial heterogeneity between fields within any one tumour section, the degree of intra-tumour heterogeneity was not related to tumour IFP (Additional File 4B-I).

Similar, more limited, analyses were carried out in the MMTV-PyMT model growing in different sites. There were no significant differences in lymphatic or blood vascular area or hypoxic fraction for MMTV-PyMT across the different sites. Proliferation was seen to vary; however, there was no relationship to tumour IFP (Additional File 5A-D).

### Imaging of perfusion in ME180 tumours growing orthotopically or in the i/m site

**Figure 6:**
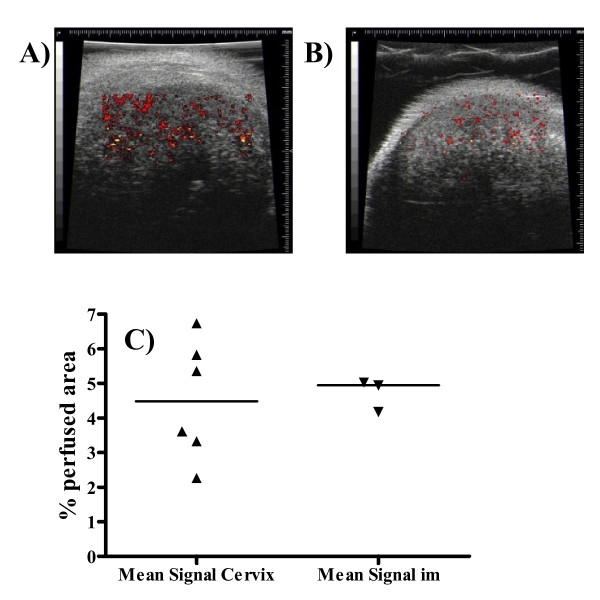
**High-frequency micro-ultrasound functional imaging**. Representative composite images of ME180 tumours growing **A) **in the cervix and **B) **i/m are shown. Positive speckle signal associated with blood flow is overlaid on top of the greyscale B mode ultrasound image. **C) **The tumour mean (from the 5 frames) percentage positive signal (blood flow) for each tumour are shown for ME180 tumours growing either in the cervix or i/m. Each point represents an individual tumour. The median is indicated on the graph.

High-frequency micro-ultrasound functional imaging and speckle variance analysis were used to detect blood flow and examine perfusion in ME180 tumours growing both orthotopically and i/m. Composite images of positive speckle signal associated with blood flow, overlaid on top of the greyscale B mode ultrasound anatomical image of the tumour, are shown for each site in Figures 6A &6B. Analysis of cineloops from five randomly selected image planes in each tumour indicated that the mean perfused area for each tumour did not differ significantly according to site (Figure 6C).

## Discussion

There is increasing interest in the adverse effects of elevated tumour IFP on drug delivery and treatment response. The aim of this study was to characterise this relatively unexplored parameter of the pathophysiological tumour microenvironment in a range of tumour models growing in different sites as a foundation for future experiments and to explore various phenotypic properties such as blood and lymphatic vascular density and tumour hypoxia that may impact on the development of tumour IFP. Systematic differences in tumour IFP as a function of both tumour type and implantation site were observed, consistent with an effect of predetermined genetic factors and interactions between the tumour and host microenvironments in regulating IFP. However, this explained only a portion of the total observed variability in IFP, possibly reflecting stochastic development and remodelling of the tumour vasculature.

IFP levels in individual tumours are influenced primarily by three fundamental pathophysiologic parameters: the trans-capillary and interstitial hydraulic conductivities and the capillary pressure [5,53,54]. In general, tumours are characterized by abnormal, highly permeable vessels and a relatively impermeable interstitium [6]. Fluid that leaks from the vessels accumulates in the interstitium and causes the pressure to rise. However, there is probably wide variability in each of these parameters among individual tumours, which contributes to heterogeneity in IFP values. In tumours where the trans-capillary conductivity is substantially less than interstitial conductivity, the IFP is low and much less than the capillary pressure. At the other extreme, where trans-capillary conductivity is high and interstitial conductivity is low, IFP becomes almost equal to the capillary pressure. This is thought to occur in many pre-clinical tumour models [55] and possibly also in human malignancies. Tumours with high flow resistance due to unregulated angiogenesis, high cell density or tumour growth in a confined space with vascular compression would be expected to have both high capillary and high interstitial pressure values. The important role of the vasculature in determining IFP is supported by studies of anti-vascular drugs that have shown reductions in tumour IFP with vascular regression or normalisation [11,39,56].

In our study, tumours grown orthotopically consistently demonstrated IFP values lower than those grown in the i/m ectopic site (Figure 1). This is in contrast to results described by Brekken et al. (2000) [30] who demonstrated higher IFP values in a human osteosarcoma line grown orthotopically as compared to s/c. This difference highlights the interaction between the tumour and the surrounding normal tissue and its influence on IFP. For example, tumours growing in bone would be expected to have high blood flow resistance and high capillary pressure because of progressive vascular compression as the cell mass increases in a confined, noncompliant space. In addition, there may be fewer normal lymphatic vessels in close proximity to the tumour, effectively reducing interstitial conductivity and driving up IFP until it equals the capillary pressure. We also demonstrated that tumours grown i/m have higher IFP values than those grown s/c, in the cervix, or in the mammary fat pads, probably reflecting similar pathophysiologic mechanisms. Overall, our results suggest that site-specific differences in vascular development and remodelling, and consequently flow resistance and pressure, as well as site-driven differences in the extracellular matrix, lead to systematic differences in IFP values. We speculate that heterogeneity in IFP values among individual tumours of the same type growing at the same site reflect the stochastic nature of unregulated angiogenesis in tumours and the random nature of the resultant vasculature.

In further support of these concepts, the MMTV-PyMT transgenic mammary gland tumours growing orthotopically in the mammary fat pad showed IFP values that were comparable to the spontaneously arising tumours. Also, there were no differences when tumours were transplanted into FVB or SCID hosts. However, when grown i/m, the IFP in the MMTV-PYMT tumours was significantly increased. Since the data showed no relationship between donor IFP and recipient IFP this suggests a site-specific effect. Similar findings in the cervical tumour models suggest individual tumour development irrespective of any tumour microenvironment-induced molecular interactions that may have existed in the original donor tumour.

It is interesting that although the tumour vasculature plays a vital role in the development of both tumour IFP and tumour hypoxia, there was no correlation between these two parameters, an effect that has also been observed both experimentally [26,57] and in the clinic [7]. In addition, there was no relationship between vascular area and IFP, with similar values being observed in all models. Furthermore, in our high frequency ultrasound speckle variance analysis of blood flow in a small number of tumours, vascular perfusion was also comparable between ME180 tumours growing in the cervix versus i/m.

There is evidence in the literature that high primary tumour IFP is associated with a higher incidence of metastatic disease [7,20,23], although Rofstad et al. (2002) suggest that it is neither necessary nor sufficient. In the present study we found no indication of a relationship between high tumour IFP and metastatic disease in any of the models in any of the sites. A controlled in vitro experiment designed to test the influence of pressure on the metastatic ability of cancer cells without the potentially confounding influence of other microenvironmental factors also showed no effect. These data are consistent with clinical data where, although a high pre-treatment IFP in cervical carcinoma patients was associated with a high risk of distant metastases following treatment with radiotherapy, there was no correlation between IFP and metastatic disease at the time of diagnosis [7]. It is possible that tumour IFP may be more a marker of treatment response than have a direct causative effect on metastatic spread.

## Conclusion

The data presented provide, for the first time, a specific analysis of tumour IFP across a selection of different tumour models encompassing human, murine transplanted and spontaneous tumours, growing in different sites. We demonstrate inherent inter-tumour heterogeneity, and a lack of correlation both between tumour donors and recipients and between different tumours growing within the same animal. Our findings indicate systematic differences in IFP as a function of tumour type consistent with predetermined inherent genetic differences that influence vascular development and the composition and organisation of the interstitium. There are also systematic differences as a function of growth site, presumably reflecting the interaction between the tumour and the surrounding host normal tissue. Nevertheless, the heterogeneity of IFP in individual tumours growing under similar conditions suggests that IFP is probably influenced to a large extent by the stochastic nature of vascular development and remodelling during tumour growth. In this context it is surprising that an analysis of various phenotypic parameters failed to show any correlation between IFP and tumour hypoxia, tumour proliferation, blood or lymphatic vascular density. Whether the lack of correlation in these parameters is indicative of no involvement in the development of tumour IFP remains uncertain, however, since the parameters are interdependent and may act together to elevate IFP. It is clear that factors influencing tumour IFP are complex and that further study is required to reveal the mechanisms that elevate IFP and its importance in therapeutic response either as a stand-alone factor or in combination with other factors.

## Competing interests

The author(s) declare that they have no competing interests.

## Authors' contributions

SJL participated in the design of the study, performed the animal experiments, analysed the IFP data and histology slides, and wrote the manuscript. TMKK was responsible for the breeding of the MMTV-PyMT transgenic mice, aided in the development of the mammary tumour surgical transplant technique, and contributed to the manuscript. AB, aided by VXY, was responsible for the analysis of the Ultrasound speckle variance data, and contributed to the manuscript. MM and RPH participated in the design of the study and were involved in writing the manuscript. All authors read and approved the final manuscript.

## Pre-publication history

The pre-publication history for this paper can be accessed here:



## Supplementary Material

Additional file 2 **Map of donor sites and corresponding recipient sites for MMTV-PyMT orthotopic mammary gland implantation. **Donor tumour fragments were taken from either the 2^nd ^or 4^th ^mammary gland. The tumour fragments were implanted into the 4^th ^mammary glands of recipient mice in one of three options: **Option 1) **Both the right and left 4^th ^mammary glands were implanted with donor fragments from the 2^nd ^mammary gland; **Option 2) **The 2^nd ^mammary gland donor was implanted in one side of the recipient animal, and the 4^th ^mammary gland donor in the other; **Option 3) **Both the right and left 4^th ^mammary glands were implanted with donor fragments from the 4^th ^mammary gland. Click here for file

Additional file 1 **Table showing tumour model IFP ranges, and mean and median values. **Table showing the range of mean tumour IFP values (mean of 3 measurements for each tumour) for each tumour model growing orthotopically and ectopically. The group mean and median IFP value and the number of tumours measured is shown (n = x). The values for tumours growing in their orthotopic site are in bold. Click here for file

Additional file 3 **Metastatic potential of tumour cells exposed to elevated pressure *in vitro***. Tumour cells were exposed to elevated pressures *in vitro *for 24, 48 or 96 hours prior to intra-venous injection *in vivo*. The lung wet weight (g) as an indication of tumour burden **(A)**, or the number of macroscopic lung lesions counted (**B-C**) is shown for each animal, grouped according to treatment group. The median is indicated for each group. **A) **KHT-C cells exposed to 20 mmHg for 24 hours, **B) **48 hours or **C) **96 hours. Click here for file

Additional file 4 **Immunohistochemical analyses of intra-tumoural heterogeneity of tumour hypoxia, proliferation and vascular area. **Analyses were carried out on ME180 tumours growing either in the cervix (n = 9) or i/m (n = 9) (see Figure 5); 10 (CD31) or 20 (EF5, Ki67, LYVE-1) random fields were analysed for each tumour section. **A) **A value for each tumour was generated as the mean percentage of positive staining [(positive pixels/total number of pixels (positive + negative)) × 100] for all fields analysed. The data are presented as median for all tumours and error bars show the range. The median is shown for each marker according to whether the tumour was grown in the cervix (n = 10) or i/m (n = 10). **B-I) **The median % positive staining and range for all 20 (or 10 for CD31) frames analysed is shown for each tumour (y-axis) plotted against tumour IFP (mmHg, x-axis); **B) **EF-5 cervix, **C) **EF5 i/m, **D) **Ki67 cervix, **E) **Ki67 i/m, **F) **CD31 cervix, **G) **CD31 i/m, **H) **LYVE-1 cervix and **I) **LYVE-1 i/m. Click here for file

Additional file 5 **Immunohistochemical analyses of tumour hypoxia, proliferation and vascular area. **Tumour sections from the PyMT tumour model growing in different sites were stained for EF5 as a marker of hypoxia, Ki67 as a marker of proliferation, CD31 as a marker of tumour vasculature and LYVE-1 as a marker of lymphatic vasculature. The median percentage of positive staining [(positive pixels/total number of pixels (positive + negative)) × 100] and the range is shown for each tumour model and site of growth; **A) **EF5 (n = 3 for all groups), **B) **Ki67 (MMTV-PyMT transgenic n = 3, MMTV-PyMT orthotopic n = 6, MMTV-PyMT i/m n = 4, **C) **CD31 (MMTV-PyMT transgenic n = 3, MMTV-PyMT orthotopic n = 8, MMTV-PyMT i/m n = 3), **D) **LYVE-1 (N = 3 for all groups). An asterix indicates significance (p ≤ 0.05). Click here for file

## References

[B1] Jain RK (1987). Transport of molecules in the tumor interstitium: a review. Cancer Res.

[B2] Jain RK (1987). Transport of molecules across tumor vasculature. Cancer Metastasis Rev.

[B3] Heldin CH, Rubin K, Pietras K, Ostman A (2004). High interstitial fluid pressure - an obstacle in cancer therapy. Nat Rev Cancer.

[B4] Baluk P, Morikawa S, Haskell A, Mancuso M, McDonald DM (2003). Abnormalities of basement membrane on blood vessels and endothelial sprouts in tumors. Am J Pathol.

[B5] Baxter LT, Jain RK (1989). Transport of fluid and macromolecules in tumors  I. Role of interstitial pressure and convection. Microvasc Res.

[B6] Milosevic M, Fyles A, Hedley D, Hill R (2004). The human tumor microenvironment: invasive (needle) measurement of oxygen and interstitial fluid pressure. Semin Radiat Oncol.

[B7] Milosevic M, Fyles A, Hedley D, Pintilie M, Levin W, Manchul L, Hill R (2001). Interstitial fluid pressure predicts survival in patients with cervix cancer independent of clinical prognostic factors and tumor oxygen measurements. Cancer Res.

[B8] Jain RK (2005). Normalization of tumor vasculature: an emerging concept in antiangiogenic therapy. Science.

[B9] Fyles A, Milosevic M, Pintilie M, Syed A, Levin W, Manchul L, Hill RP (2006). Long-term performance of interstial fluid pressure and hypoxia as prognostic factors in cervix cancer. Radiother Oncol.

[B10] Eikenes L, Bruland OS, Brekken C, Davies Cde L (2004). Collagenase increases the transcapillary pressure gradient and improves the uptake and distribution of monoclonal antibodies in human osteosarcoma xenografts. Cancer Res.

[B11] Pietras K, Ostman A, Sjoquist M, Buchdunger E, Reed RK, Heldin CH, Rubin K (2001). Inhibition of platelet-derived growth factor receptors reduces interstitial hypertension and increases transcapillary transport in tumors. Cancer Res.

[B12] Pietras K, Stumm M, Hubert M, Buchdunger E, Rubin K, Heldin CH, McSheehy P, Wartmann M, Ostman A (2003). STI571 enhances the therapeutic index of epothilone B by a tumor-selective increase of drug uptake. Clin Cancer Res.

[B13] Pietras K, Rubin K, Sjoblom T, Buchdunger E, Sjoquist M, Heldin CH, Ostman A (2002). Inhibition of PDGF receptor signaling in tumor stroma enhances antitumor effect of chemotherapy. Cancer Res.

[B14] Rubin K, Sjoquist M, Gustafsson AM, Isaksson B, Salvessen G, Reed RK (2000). Lowering of tumoral interstitial fluid pressure by prostaglandin E(1) is paralleled by an increased uptake of (51)Cr-EDTA. Int J Cancer.

[B15] Salnikov AV, Iversen VV, Koisti M, Sundberg C, Johansson L, Stuhr LB, Sjoquist M, Ahlstrom H, Reed RK, Rubin K (2003). Lowering of tumor interstitial fluid pressure specifically augments efficacy of chemotherapy. Faseb J.

[B16] Netti PA, Baxter LT, Boucher Y, Skalak R, Jain RK (1995). Time-dependent behaviour of interstitial fluid pressure in solid tumors: implications for drug delivery. Cancer Res.

[B17] Netti PA, Hamberg LM, Babich JW, Kierstead D, Graham W, Hunter GJ, Wolf GL, Fischman A, Boucher Y, Jain RK (1999). Enhancement of fluid filtration across tumor vessels: implication for delivery of macromolecules. Proc Natl Acad Sci U S A.

[B18] Roh HD, Kalnicki S, Buchsbaum R, Bloomer WD, Jain RK (1991). Interstitial hypertension in carcinoma of uterine cervix in patients:  Possible correlation with tumor oxygenation and radiation response.. Cancer Res.

[B19] Curti BD, Urba WJ, Alvord WG, Janik JE, Smith JW, Madara K, Longo DL (1993). Interstitial pressure of subcutaneous nodules in melanoma and lymphoma patients: changes during treatment. Cancer Res.

[B20] Rofstad EK, Tunheim SH, Mathiesen B, Graff BA, Halsor EF, Nilsen K, Galappathi K (2002). Pulmonary and lymph node metastasis is associated with primary tumor interstitial fluid pressure in human melanoma xenografts. Cancer Res.

[B21] Less JR, Posner MC, Boucher Y, Borochovitz D, Wolmark N, Jain RK (1992). Interstitial hypertension in human breast and colorectal tumors. Cancer Res.

[B22] Taghian AG, Abi-Raad R, Assaad SI, Casty A, Ancukiewicz M, Yeh E, Molokhia P, Attia K, Sullivan T, Kuter I, Boucher Y, Powell SN (2005). Paclitaxel decreases the interstitial fluid pressure and improves oxygenation in breast cancers in patients treated with neoadjuvant chemotherapy: clinical implications. J Clin Oncol.

[B23] Nathanson SD, Nelson L (1994). Interstitial fluid pressure in breast cancer, benign breast conditions, and breast parenchyma. Ann Surg Oncol.

[B24] Boucher Y, Kirkwood JM, Opacic D (1991). Interstitial hypertension in superficial metastatic melanomas in humans. Cancer Res.

[B25] DiResta GR, Lee J, Larson SM, Arbit E (1993). Characterization of neuroblastoma xenograft in rat flank. I. Growth, interstitial fluid pressure, and interstitial fluid velocity distribution profiles. Microvasc Res.

[B26] Boucher Y, Lee I, Jain RK (1995). Lack of general correlation between interstitial fluid pressure and oxygen partial pressure in solid tumors. Microvasc Res.

[B27] Hori K, Suzuki M, Saito S, Tanda S, Zhang QH, Li HC (1994). Changes in vessel pressure and interstitial fluid pressure of normal subcutis and subcutaneous tumor in rats due to angiotensin II. Microvasc Res.

[B28] Znati CA, Rosenstein M, Boucher Y, Epperly MW, Bloomer WD, Jain RK (1996). Effect of radiation on interstitial fluid pressure and oxygenation in a human tumor xenograft. Cancer Res.

[B29] Kerbel RS, Cornil I, Theodorescu D (1991). Importance of orthotopic transplantation procedures in assessing the effects of transfected genes on human tumor growth and metastasis. Cancer Metastasis Rev.

[B30] Brekken C, Bruland OS, de Lange Davies C (2000). Interstitial fluid pressure in human osteosarcoma xenografts: significance of implantation site and the response to intratumoral injection of hyaluronidase. Anticancer Res.

[B31] Cairns RA, Hill RP (2004). Acute hypoxia enhances spontaneous lymph node metastasis in an orthotopic murine model of human cervical carcinoma. Cancer Res.

[B32] Cairns RA, Kalliomaki T, Hill RP (2001). Acute (cyclic) hypoxia enhances spontaneous metastasis of KHT murine tumors. Cancer Res.

[B33] Guy CT, Cardiff RD, Muller WJ (1992). Induction of mammary tumors by expression of polyomavirus middle T oncogene: a transgenic mouse model for metastatic disease. Mol Cell Biol.

[B34] Hagendoorn J, Tong R, Fukumura D, Lin Q, Lobo J, Padera TP, Xu L, Kucherlapati R, Jain RK (2006). Onset of abnormal blood and lymphatic vessel function and interstitial hypertension in early stages of carcinogenesis. Cancer Res.

[B35] Bristow RG, Hardy PA, Hill RP (1990). Comparison between in vitro radiosensitivity and in vivo radioresponse of murine tumor cell lines. I: Parameters of in vitro radiosensitivity and endogenous cellular glutathione levels. Int J Radiat Oncol Biol Phys.

[B36] Cairns RA, Hill RP (2004). A fluorescent orthotopic model of metastatic cervical carcinoma. Clin Exp Metastasis.

[B37] Young SD, Marshall RS, Hill RP (1988). Hypoxia induces DNA overreplication and enhances metastatic potential of murine tumor cells. Proc Natl Acad Sci U S A.

[B38] Fadnes HO, Reed RK, Aukland K (1977). Interstitial fluid pressure in rats measured with a modified wick technique. Microvasc Res.

[B39] Skliarenko JV, Lunt SJ, Gordon ML, Vitkin A, Milosevic M, Hill RP (2006). Effects of the vascular disrupting agent ZD6126 on interstitial fluid pressure and cell survival in tumors. Cancer Res.

[B40] Franco M, Man S, Chen L, Emmenegger U, Shaked Y, Cheung AM, Brown AS, Hicklin DJ, Foster FS, Kerbel RS (2006). Targeted anti-vascular endothelial growth factor receptor-2 therapy leads to short-term and long-term impairment of vascular function and increase in tumor hypoxia. Cancer Res.

[B41] Yang VXD, Needles A, Vray D (2004). High frequency ultrasound speckle flow imaging: comparison with doppler optical coherence tomography (DOCT).. Proc IEEE Ultrasound Symp.

[B42] Gutmann R, Leunig M, Feyh J, Goetz AE, Messmer K, Kastenbauer E, Jain RK (1992). Interstitial hypertension in head and neck tumors in patients: correlation with tumor size. Cancer Res.

[B43] Podobnik B, Sersa G, Miklavcic D (2001). Effect of hydralazine on interstitial fluid pressure in experimental tumours and in normal tissue. In Vivo.

[B44] Tufto I, Rofstad EK (1999). Interstitial fluid pressure and capillary diameter distribution in human melanoma xenografts. Microvasc Res.

[B45] Tufto I, Rofstad EK (1995). Interstitial fluid pressure in human melanoma xenografts. Relationship to fractional tumor water content, tumor size, and tumor volume-doubling time. Acta Oncol.

[B46] Rofstad EK, Mathiesen B, Henriksen K, Kindem K, Galappathi K (2005). The tumor bed effect: increased metastatic dissemination from hypoxia-induced up-regulation of metastasis-promoting gene products. Cancer Res.

[B47] De Jaeger K, Kavanagh MC, Hill RP (2001). Relationship of hypoxia to metastatic ability in rodent tumours. Br J Cancer.

[B48] Rofstad EK, Danielsen T (1999). Hypoxia-induced metastasis of human melanoma cells: involvement of vascular endothelial growth factor-mediated angiogenesis. Br J Cancer.

[B49] Rofstad EK, Sundfor K, Lyng H, Trope CG (2000). Hypoxia-induced treatment failure in advanced squamous cell carcinoma of the uterine cervix is primarily due to hypoxia-induced radiation resistance rather than hypoxia-induced metastasis. Br J Cancer.

[B50] Rofstad EK, Halsor EF (2002). Hypoxia-associated spontaneous pulmonary metastasis in human melanoma xenografts: involvement of microvascular hot spots induced in hypoxic foci by interleukin 8. Br J Cancer.

[B51] Diresta GR, Nathan SS, Manoso MW, Casas-Ganem J, Wyatt C, Kubo T, Boland PJ, Athanasian EA, Miodownik J, Gorlick R, Healey JH (2005). Cell proliferation of cultured human cancer cells are affected by the elevated tumor pressures that exist in vivo. Ann Biomed Eng.

[B52] Nathan SS, DiResta GR, Casas-Ganem JE, Hoang BH, Sowers R, Yang R, Huvos AG, Gorlick R, Healey JH (2005). Elevated physiologic tumor pressure promotes proliferation and chemosensitivity in human osteosarcoma. Clin Cancer Res.

[B53] Baxter LT, Jain RK, Svensjo E (1987). Vascular permeability and interstitial diffusion of macromolecules in the hamster cheek pouch: effects of vasoactive drugs. Microvasc Res.

[B54] Jain RK, Tong RT, Munn LL (2007). Effect of vascular normalization by antiangiogenic therapy on interstitial hypertension, peritumor edema, and lymphatic metastasis: insights from a mathematical model. Cancer Res.

[B55] Boucher Y, Baxter LT, Jain RK (1990). Interstitial pressure gradients in tissue isolated and subcutaneous tissues: implications for therapy. Cancer Res.

[B56] Tong RT, Boucher Y, Kozin SV, Winkler F, Hicklin DJ, Jain RK (2004). Vascular normalization by vascular endothelial growth factor receptor 2 blockade induces a pressure gradient across the vasculature and improves drug penetration in tumors. Cancer Res.

[B57] Tufto I, Lyng H, Rofstad EK (1996). Interstitial fluid pressure, perfusion rate and oxygen tension in human melanoma xenografts. Br J Cancer Suppl.

