# Supplementary figures and images for: Interstitial fluid pressure, vascularity and metastasis in ectopic, orthotopic and spontaneous tumours
Source: BMC Cancer. 2008 Jan 7;8:2. doi: 10.1186/1471-2407-8-2 (PMC2245966; doi:10.1186/1471-2407-8-2)

## Additional File 2

**Option 1)**

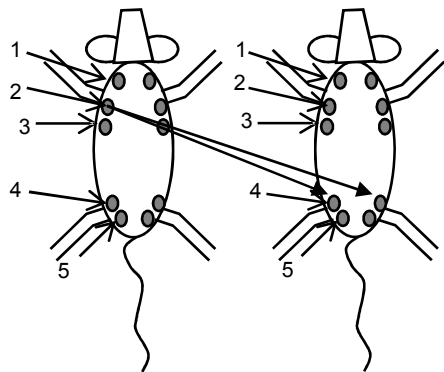

**Option 2)**

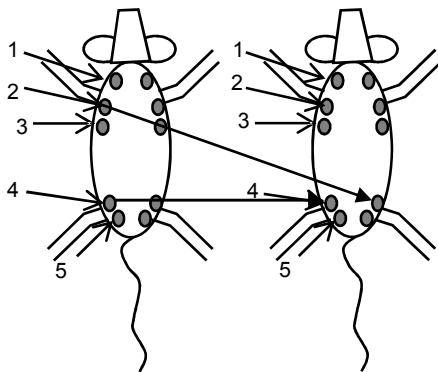

**Option 3)**

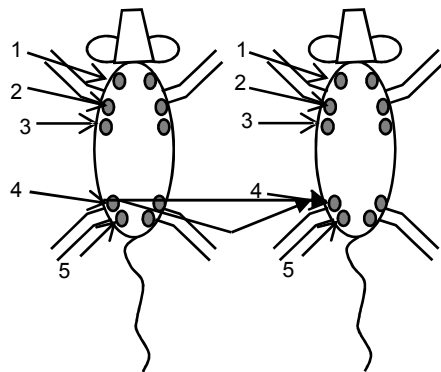

Supplement: Additional file 2 — Map of donor sites and corresponding recipient sites for MMTV-PyMT orthotopic mammary gland implantation. Donor tumour fragments were taken from either the 2nd or 4th mammary gland. The tumour fragments were implanted into the 4th mammary glands of recipient mice in one of three options: Option 1) Both the right and left 4th mammary glands were implanted with donor fragments from the 2nd mammary gland; Option 2) The 2nd mammary gland donor was implanted in one side of the recipient animal, and the 4th mammary gland donor in the other; Option 3) Both the right and left 4th mammary glands were implanted with donor fragments from the 4th mammary gland. [file 1471-2407-8-2-S2.pdf]

# Additional File 3

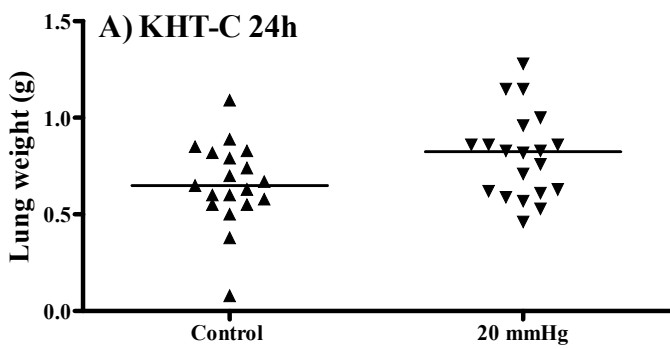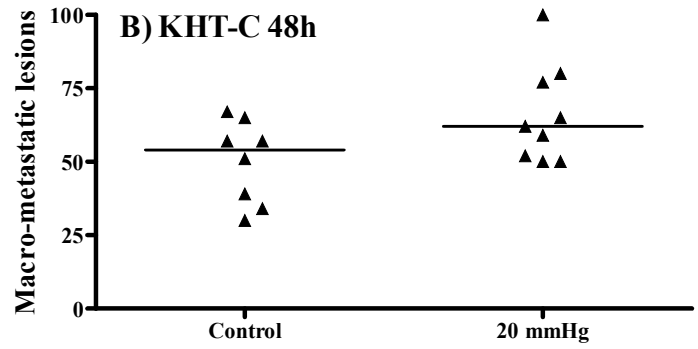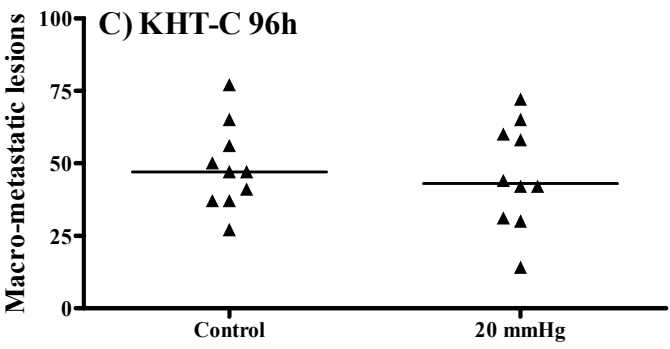

Supplement: Additional file 3 — Metastatic potential of tumour cells exposed to elevated pressure in vitro. Tumour cells were exposed to elevated pressures in vitro for 24, 48 or 96 hours prior to intra-venous injection in vivo. The lung wet weight (g) as an indication of tumour burden (A), or the number of macroscopic lung lesions counted (B-C) is shown for each animal, grouped according to treatment group. The median is indicated for each group. A) KHT-C cells exposed to 20 mmHg for 24 hours, B) 48 hours or C) 96 hours. [file 1471-2407-8-2-S3.pdf]

Additional File 4

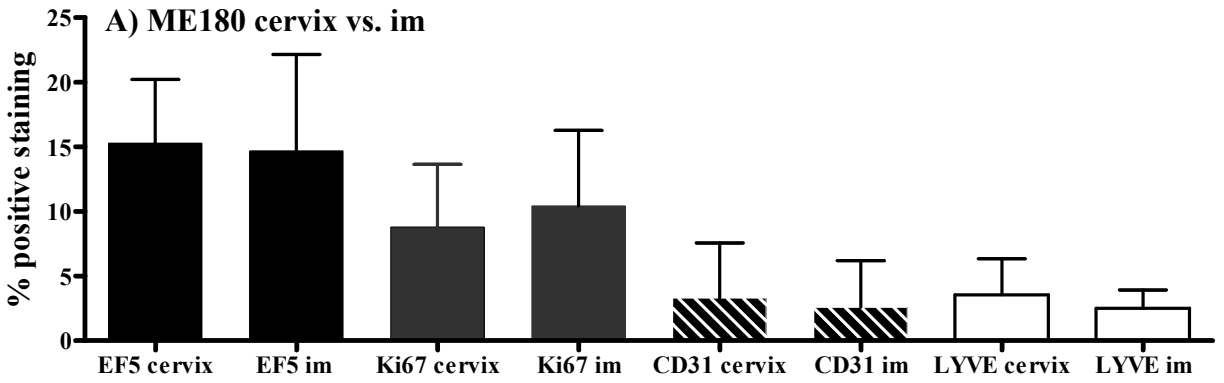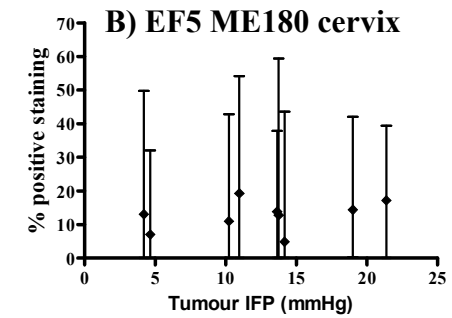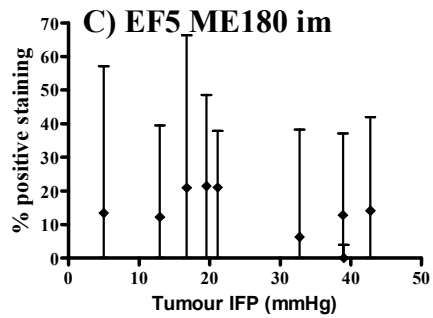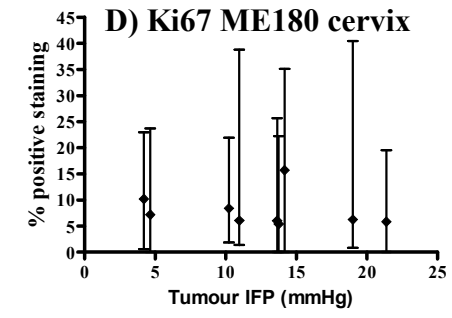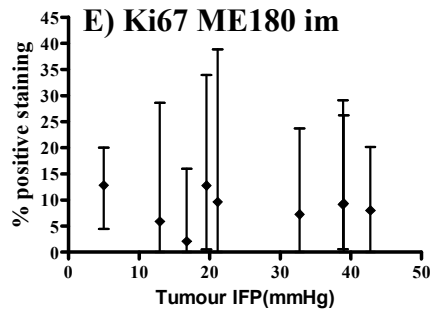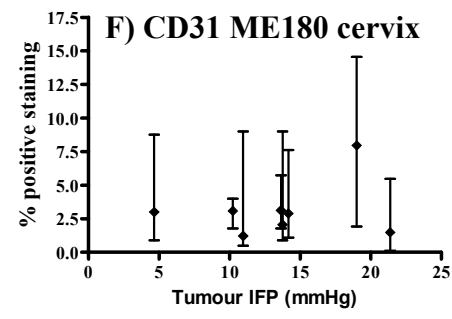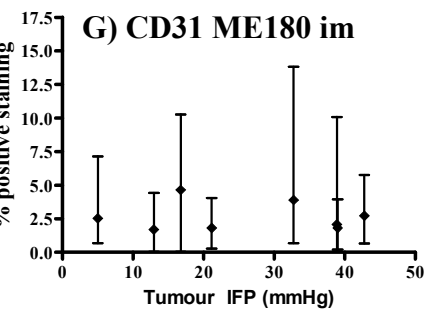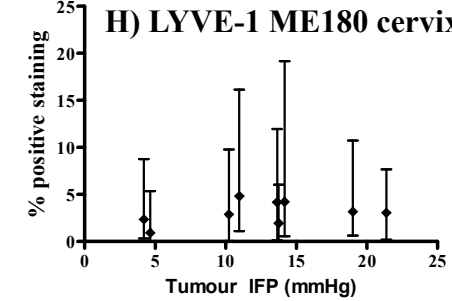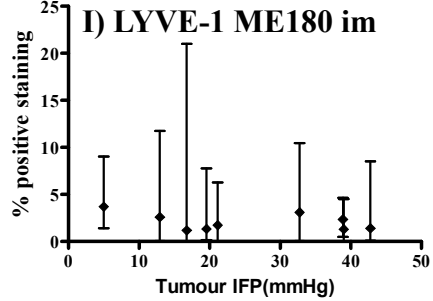

Supplement: Additional file 4 — Immunohistochemical analyses of intra-tumoural heterogeneity of tumour hypoxia, proliferation and vascular area. Analyses were carried out on ME180 tumours growing either in the cervix (n = 9) or i/m (n = 9) (see Figure 5); 10 (CD31) or 20 (EF5, Ki67, LYVE-1) random fields were analysed for each tumour section. A) A value for each tumour was generated as the mean percentage of positive staining [(positive pixels/total number of pixels (positive + negative)) × 100] for all fields analysed. The data are presented as median for all tumours and error bars show the range. The median is shown for each marker according to whether the tumour was grown in the cervix (n = 10) or i/m (n = 10). B-I) The median % positive staining and range for all 20 (or 10 for CD31) frames analysed is shown for each tumour (y-axis) plotted against tumour IFP (mmHg, x-axis); B) EF-5 cervix, C) EF5 i/m, D) Ki67 cervix, E) Ki67 i/m, F) CD31 cervix, G) CD31 i/m, H) LYVE-1 cervix and I) LYVE-1 i/m. [file 1471-2407-8-2-S4.pdf]

## Additional File 5

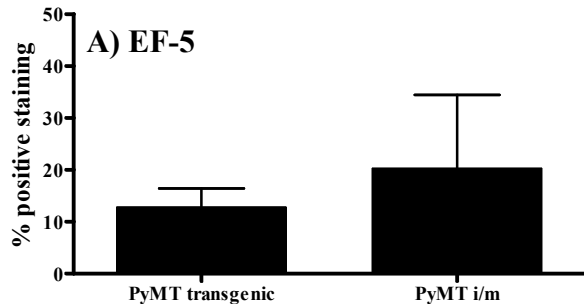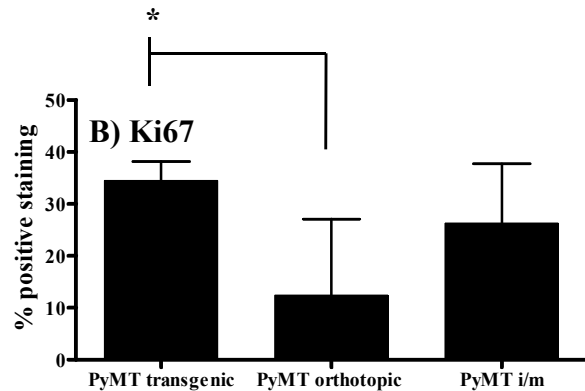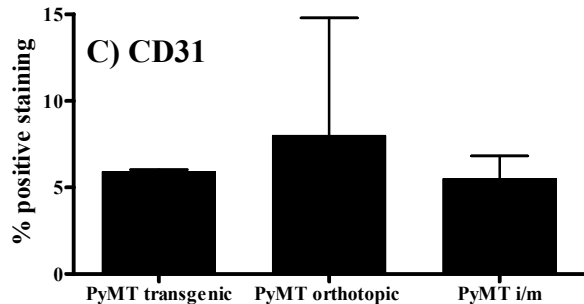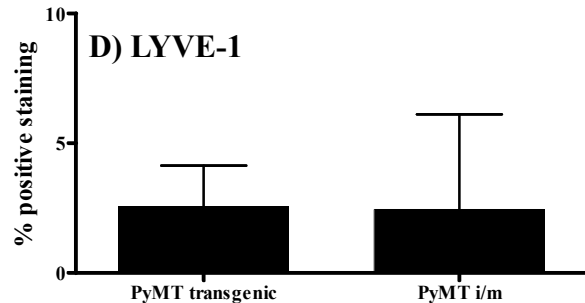

Supplement: Additional file 5 — Immunohistochemical analyses of tumour hypoxia, proliferation and vascular area. Tumour sections from the PyMT tumour model growing in different sites were stained for EF5 as a marker of hypoxia, Ki67 as a marker of proliferation, CD31 as a marker of tumour vasculature and LYVE-1 as a marker of lymphatic vasculature. The median percentage of positive staining [(positive pixels/total number of pixels (positive + negative)) × 100] and the range is shown for each tumour model and site of growth; A) EF5 (n = 3 for all groups), B) Ki67 (MMTV-PyMT transgenic n = 3, MMTV-PyMT orthotopic n = 6, MMTV-PyMT i/m n = 4, C) CD31 (MMTV-PyMT transgenic n = 3, MMTV-PyMT orthotopic n = 8, MMTV-PyMT i/m n = 3), D) LYVE-1 (N = 3 for all groups). An asterix indicates significance (p ≤ 0.05). [file 1471-2407-8-2-S5.pdf]
