# Supplementary material for: Interstitial fluid pressure, vascularity and metastasis in ectopic, orthotopic and spontaneous tumours
Source: BMC Cancer. 2008 Jan 7;8:2. doi: 10.1186/1471-2407-8-2 (PMC2245966; doi:10.1186/1471-2407-8-2)
Supplement: Additional file 1 — Table showing tumour model IFP ranges, and mean and median values. Table showing the range of mean tumour IFP values (mean of 3 measurements for each tumour) for each tumour model growing orthotopically and ectopically. The group mean and median IFP value and the number of tumours measured is shown (n = x). The values for tumours growing in their orthotopic site are in bold. [file 1471-2407-8-2-S1.pdf]

Additional File 1 - Table showing tumour model IFP ranges, and mean and median values.

	Sub-cutaneously	Intra-muscularly	Cervix	Spontaneous MG	Transplanted MG
**KHT-C**	Range: 1-14 mmHg<br>Mean: 6 mmHg<br>Median: 6 mmHg<br>(n=12)	**Range: 2-42 mmHg**<br>**Mean: 16 mmHg**<br>**Median: 14 mmHg**<br>**(n=104)**			
**Me180**		Range: 5-68 mmHg<br>Mean: 33 mmHg<br>Median: 30 mmHg<br>(n=26)	**Range: 4-21 mmHg**<br>**Mean: 9 mmHg**<br>**Median: 8 mmHg**<br>**(n=28)**		
**SiHa**		Range: 20-56 mmHg<br>Mean: 32 mmHg<br>Median: 28 mmHg<br>(n=9)	**Range: 2-26 mmHg**<br>**Mean: 14 mmHg**<br>**Median: 15 mmHg**<br>**(n=10)**		
**MMTV-PyMT**		Range: 13-45 mmHg<br>Mean: 26 mmHg<br>Median: 25 mmHg<br>(n=15)		**Range: 2-20 mmHg**<br>**Mean: 8 mmHg**<br>**Median: 7 mmHg**<br>**(n=27)**	**Range: 2-22 mmHg**<br>**Mean: 9 mmHg**<br>**Median: 8 mmHg**<br>**(n=62)**
